# The Agreement between Fat_max_ and the Gas-Exchange and Blood Lactate Thresholds: A Systematic Review and Meta-Analysis

**DOI:** 10.1249/MSS.0000000000004027

**Published:** 2026-05-18

**Authors:** CLORINDA HOGAN, TROY J. CROSS, MEGAN TIONG, PRADEEP PRATHEEPAN, ANNELIESE BLAXLAND, ELIZABETH MACHAN, KIERON ROONEY

**Affiliations:** 1 Discipline of Exercise and Sport Science, Faculty of Medicine and Health, The University of Sydney, Camperdown, NSW, AUSTRALIA; 2 Heat and Health Research Centre, Faculty of Medicine and Health, The University of Sydney, Camperdown, AUSTRALIA; 3 CIRUS Centre for Sleep and Chronobiology, Woolcock Institute of Medical Research, Sydney, AUSTRALIA

**Keywords:** EXERCISE TESTING, FUEL UTILIZATION, INDIRECT CALORIMETRY, LACTATE, MAXIMAL FAT OXIDATION, VENTILATORY THRESHOLD

## Abstract

**Background:**

The relationship between the exercise intensity at which maximal fat oxidation occurs (Fat_max_) and the lactate threshold/gas-exchange threshold (LT/GET) has gained interest in both sport performance and health-related communities, yet the absolute agreement between these two “thresholds” remains inconsistent. This review aims to systematically assess the available literature to determine whether Fat_max_ occurs at the same exercise intensity as the LT/GET and to examine whether aspects of study design moderated this relationship.

**Methods:**

PubMed, MEDLINE, Embase, and Scopus were systematically searched from inception to January 20, 2025. Studies were included if they reported Fat_max_ and measures of the LT/GET in healthy adults. Exercise intensities corresponding to these thresholds were analyzed using a random-effects multilevel meta-analysis. Univariate moderator analyses were performed to identify potential methodological or participant-related moderators of the effect size.

**Results:**

The meta-analysis (*N* = 42 studies) revealed that Fat_max_ occurred at a significantly lower exercise intensity than LT/GET, by approximately 5.78% $\dot{V}O_{2max}$, 4.75 mL·min^−1^·kg^−1^ $\dot{V}O_{2}$, 22 W, or 11 beats ·min^−1^ (effect size = 0.74, 95% confidence interval = 0.32–1.16, *P* = 0.001). Moderator analyses indicated that differences between Fat_max_ and LT/GET varied according to participant characteristics (e.g., age, sex, and fitness level), nutritional status, study design, testing protocols, and analytical methods.

**Conclusions:**

Although Fat_max_ typically occurs at a lower exercise intensity than the LT/GET, this difference appears smaller in sedentary individuals (<20 yr), when testing is performed with a <6-h fasting period, and when treadmill-based, two separate sessions protocols are employed. Under these conditions, the LT/GET could serve as a surrogate for Fat_max_ when direct substrate oxidation measurement is not feasible.

The absolute rate of fat oxidation increases with exercise intensity from low- to moderate-intensity exercise (~25%–65% maximal oxygen consumption [$\dot{V}O_{2max}$]), before declining rapidly at higher intensities (i.e., ~85%–95% $\dot{V}O_{2max}$) (1–3). Thus, at an intermediate submaximal exercise intensity, there occurs a point of maximal fat oxidation (MFO). The exercise intensity corresponding to MFO is termed the Fat_max_ (2). Training at an individual’s Fat_max_ elicits physiological and metabolic adaptations, enhancing fat oxidation capacity, endurance performance, and overall metabolic health (4–9). Certainly, training at this exercise intensity elicits a 44% increase in fat oxidation rates, compared with no changes when interval training is performed (5). Furthermore, endurance training at Fat_max_ improves body composition (6–9),$\dot{V}O_{2max}$ (6–8), insulin sensitivity (6,8,9), and lipid profiles (7,8) in both pediatric and adult clinical populations.

Seeing that the capacity to oxidize fat during exercise has been associated with endurance performance success (10,11) and markers of metabolic health (12), the ability to identify Fat_max_ in a time-efficient manner has become increasingly important (5). Some researchers have suggested that Fat_max_ can be estimated from a single, graded exercise test using the moderate-to-heavy intensity transition as a surrogate (13–15). Whereas other investigators have observed significant differences between this exercise-intensity transition and Fat_max,_ suggesting these two “thresholds” should be determined using separate protocols (16). The lactate threshold (LT), the gas-exchange threshold (GET), and the ventilatory threshold (VT) are well-established markers of the moderate-to-heavy intensity transition (17,18); therefore, this transition point will be referred to as the LT/GET throughout (19).

A recent meta-analysis (20) including 14 studies reported no significant differences between Fat_max_ (effect size [ES] = −0.23, 95% confidence interval [CI] = −0.56 to 0.09, *P* > 0.05) and markers of the moderate-to-heavy intensity transition (i.e., first lactate threshold [LT_1_], GET, or first ventilatory threshold ). A follow-up review from the same group was conducted to assess the agreement between Fat_max_ and LT/GET, with LT/GET intensities frequently falling outside the predefined ±10% MFO range (21). Although a moderate association was observed, no consistent directional bias was identified, indicating that LT/GET cannot reliably serve as a surrogate for Fat_max_ at the individual level. However, the results must be interpreted with caution, given that the authors systematically searched only two databases (MEDLINE and Google Scholar) using a date range spanning from January 2001 to April 2021 (i.e., most recent 20 yr). Despite the technical term “Fat_max_” not being introduced until 2001 (22), researchers have measured peak fat oxidation during exercise before this year. For example, Romijn et al. (3) and Astorino (23) examined fat and carbohydrate oxidation across varying exercise intensities. The inclusion criteria stated by Peric et al. (20) were that studies must report Fat_max_, LT/GET, and the statistical correlation between those two parameters, resulting in the potential exclusion of studies where the two thresholds were not primary outcomes, nor were correlations between these two variables performed.

In turn, it remains unknown whether a more comprehensive search of the literature (since inception across multiple databases) would yield a greater number of eligible studies for inclusion in a meta-analysis. Therefore, this systematic review aimed to create a synthesis from existing literature and determine whether Fat_max_ occurs at the same intensity as LT/GET within healthy participants. It is clear from a preliminary search of the literature that studies measuring both thresholds have included a wide range of participant preparatory conditions (13,16,24), acute nutritional status conditions (23,25), exercise modes and protocols (15,26,27), and data analysis methods (15,28). Therefore, a secondary aim was to determine whether various aspects of study design were moderators of the reported ES between exercise intensities corresponding to Fat_max_ and LT/GET.

## METHODS

This systematic review and meta-analysis was performed under the Preferred Reporting Items for Systematic Reviews and Meta-Analyses (PRISMA) guidelines (29). The protocol for this review was registered with PROSPERO (ID CRD42022346457).

### Search strategy

Four electronic databases (PubMed, Medline, Embase, and Scopus) were systematically searched from inception to the July 9, 2022, with an update on January 20, 2025. The search strategy involved combining the following keywords: “lactate” OR “ventilat*” OR “gas exchange threshold” OR “metabolic threshold” OR “aerobic threshold” OR “anerobic threshold” OR “anaerobic threshold” AND “fat max” OR “Fatmax” OR “maximal fat oxidation” OR “peak fat oxidation” OR “lipomax” OR “fuel utili*.”

### Eligibility criteria

Studies were considered eligible for inclusion if they: (1) were original research articles published in peer-reviewed journals; (2) recruited nonobese (body mass index < 30 kg·m^−2^) ostensibly healthy human participants without known cardiometabolic diseases, cancer, or receiving medications affecting substrate metabolism; (3) were cross-sectional by design or, in the case of intervention studies, included a control group or baseline measurement time point; (4) assessed the moderate-to-heavy intensity transition during graded exercise via any of its surrogate markers, including blood or plasma lactate, GET or VT; (5) provided an assessment of Fat_max_ during a graded exercise protocol; and if they (6) reported the mean, and a measure of dispersion around the mean (i.e., standard deviation [SD]; or standard error [SE]) for the exercise intensities corresponding to LT/GET and Fat_max_.

If descriptives were not reported in the study, the corresponding data were requested by contacting the study authors. Studies were excluded if either the LT, GET, and VT, Fat_max_, or $\dot{V}O_{2max}$ were obtained via prediction equations, reference values, and/or extrapolated from submaximal exercise data. To facilitate comparison between LT/GET and Fat_max_, studies were only included if each threshold was reported in identical units of exercise intensity. Acceptable units of exercise intensity were: $\dot{V}O_{2}$ reported in absolute (L·min^−1^) or relative (e.g., mL·kg^−1^·min^−1^) units or expressed as a percent of $\dot{V}O_{2max}$; work rates reported in absolute (W) and relative (e.g., W·kg^−1^) units, or expressed as a percent of maximum W (%W_max_); and heart rate (HR) in absolute (beats·min^−1^) units or expressed as a percentage of maximum HR (%HR_max_) or HR reserve (HRR). Studies that calculated and defined LT as 4 mmol·L^−1^ were excluded, given that this discrete point has been shown to represent the maximal lactate steady state (MLSS) (30,31).

### Study screening and selection process

Search results were exported from each database into EndNote reference management software (EndNote 20.3, Clarivate, Philadelphia), and then imported into Covidence systematic review software (Veritas Health Innovations, Melbourne, Australia) (32). Duplicates were removed, and each title and abstract were screened independently by four reviewers (C. H., K. R., T. J. C., and M. T.) according to the eligibility criteria. In the case of conflict, a third reviewer (K. R. and T. J. C.) decided, and consensus was reached in all cases. Full-text copies of each included study were obtained and further screened by five reviewers (C. H., K. R., T. J. C., A. B., are P. P.), and any conflicts were resolved by a third reviewer (K.R., or T.J.C.).

### Data extraction

Three authors (C. H., M. T., and P. P.) independently extracted data using a customized Microsoft Excel spreadsheet (see Supplementary Material 1, Supplemental Digital Content 1, https://links.lww.com/MSS/D419). Descriptive statistics were recorded, including means and SD. In the case where a study included a clinical population, only the data from the healthy control group were extracted. If a study implemented an intervention of any kind, only preintervention data were extracted.

### Risk of bias assessment

Risk of bias was assessed by using the relevant tool depending on the study design that was agreed upon between three independent reviewers (C. H., M. T., and P. P.), and any discrepancies were resolved with a third reviewer (M. T. and P. P.). Interrater reliability between reviewers was assessed using Cohen Kappa coefficient (κ). Results are displayed graphically using the *Robvis* statistical package in R language and environment for statistical computing (Version 4.3.1) (33). See Supplementary Material 2, Supplemental Digital Content 2, https://links.lww.com/MSS/D420, for specific details on tools used for each study design.

### Multilevel meta-analysis

A random-effects multilevel meta-analysis was performed in R using the *meta*, *metafor,* and *dmetar* statistical packages. The hierarchical structure nested in the three-level model was: (1) each participant within a sample; (2) within-study heterogeneity (i.e., each ES); and (3) between-study heterogeneity (i.e., each study). Model parameters were calculated using the restricted maximum likelihood estimation. The standardized mean difference (SMD) using Hedge’s G was used to calculate the pooled ES and associated 95% CIs. A forest plot was generated to visually depict the studies' pooled SMD and associated 95% CI. The heterogeneity across levels was assessed using a multilevel version of the *I^2^* test, where ≤25% was low, ≤50% moderate, and >75% was considered high heterogeneity (34). Funnel plots, contoured enhanced funnel plots, Egger’s regression test (35), and the trim and fill procedure (36) were used to consider publication bias. Sensitivity analysis was performed using the leave-one-out method (37), whereby each study was sequentially removed and the meta-analysis re-run to assess the influence of individual studies on the overall effect. Univariate moderator analyses relating to participant characteristics, participant acute nutritional status, study design, protocol characteristics and equipment, and analysis methods were performed to separately explore whether each moderator influenced the pooled ES for the exercise intensities corresponding to Fat_max_ and LT/GET. Statistical analyses were considered significant if *P* < 0.05. A detailed description of the statistical analyses performed in this study is provided in Supplementary Material 3, Supplemental Digital Content 2, https://links.lww.com/MSS/D420.

## RESULTS

### Study selection

**FIGURE 1:**
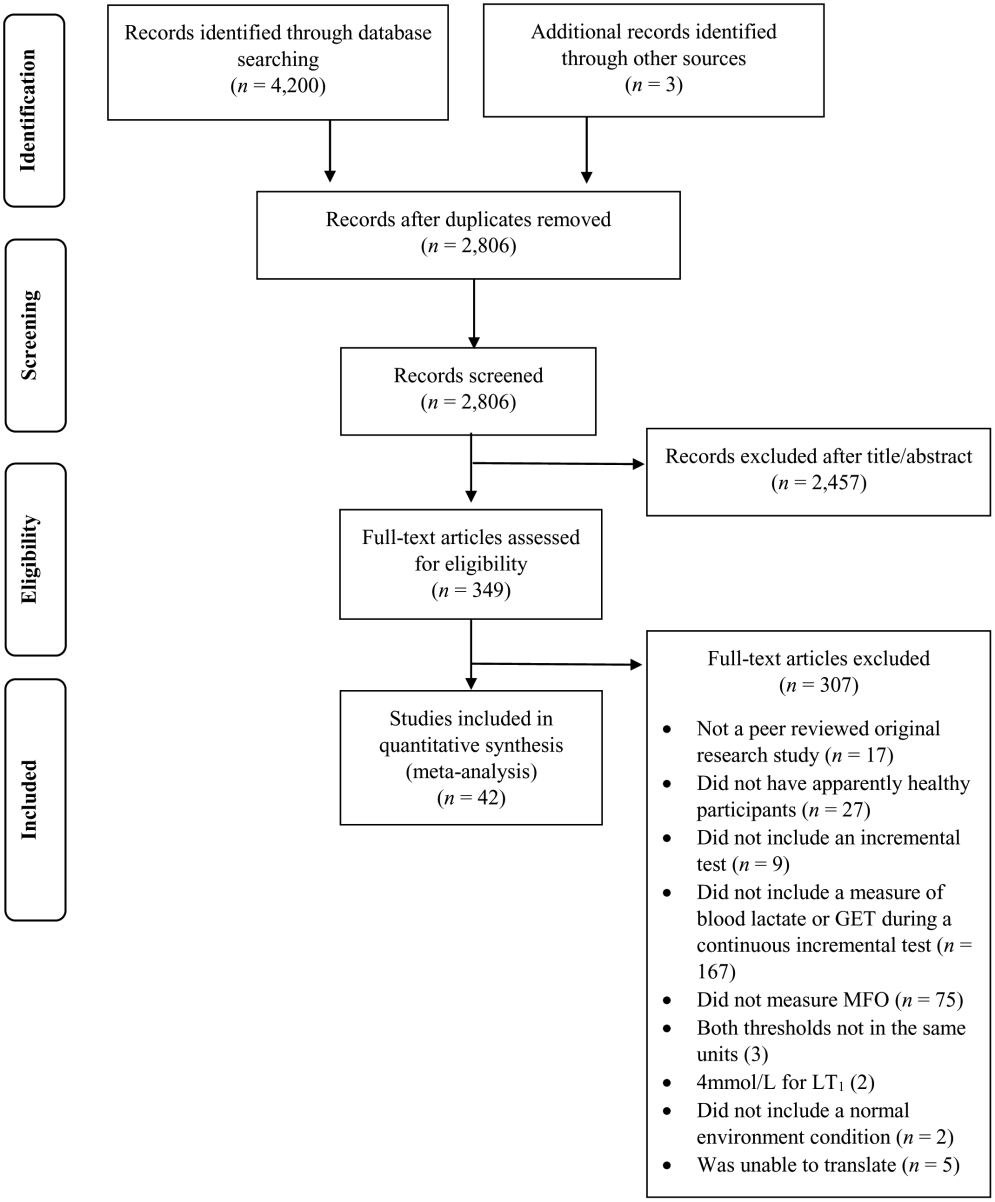
PRISMA flow diagram summarizing study identification and selection process.

The systematic identification, screening, and selection process of relevant literature are summarized in Figure 1. There were 66 potential studies identified. Of these studies, a total of 34 authors were contacted for provision of incomplete data, and 10 authors responded. Thus, 24 studies were excluded from further analysis. In total, 42 studies were included in our systematic review, which, in turn, yielded a total of 158 estimates of ES (e.g., median number of ES was 2 per study).

### Study and participant characteristics

The included studies are summarized in Supplementary Materials 4, Supplemental Digital Content 2, https://links.lww.com/MSS/D420 and Supplementary Materials 5, Supplemental Digital Content 3, https://links.lww.com/MSS/D450. The included studies ranged across 18 different countries, the majority being from Europe (*n* = 27, 64%). There were 22 studies (52%) that included a male-only sample, 13 studies (29%) that included both sexes, and only three studies (7%) that included female-only samples. There was a total of 1505 participants, of which 101 participants’ sex were not recorded. Of the remaining participants, there were 958 males (68%), 402 females (29%), and 44 (3%) were reported as combined males and females. Mean age ranged from 13.9 to 48.5 yr. The average age of participants of the included studies was distributed as follows: 15% were <20 yr, 40% between 20 and 29 yr, 41% between 30 and 39 yr, and 4% between 40 and 49 yr. Across the included participants (*N* = 1505), the pooled weighted mean age was 28.1 ± 10.9 yr. We assigned a participant fitness category to each study according to the author descriptions of the physical activity and exercise levels of their participants, and any instances where participants’ $\dot{V}O_{2max}$ were compared with normative values according to sex, age, and exercise modality (38). Accordingly, participants' fitness level comprised of: elite = 448 participants (30%), trained = 525 participants (35%), active = 407 participants (27%), sedentary = 125 participants (8%).

### Acute nutritional status

Fasting time was specified in 32 studies (76%) and ranged from 2 h on the morning of testing to an overnight fast of 10–12 h. Most studies (*n* = 20, 48%) implemented an overnight fast of 10–12 or 7–10 h. In 10 studies (24%), it was unclear what fasting protocol was implemented before the exercise tests. Participants were instructed to maintain their regular dietary habits in 19 studies (45%). In 12 studies (29%), participants consumed a carbohydrate-rich diet ranging from the dinner before 3 d pretesting. Standardized meals were prescribed by a nutritionist (*n* = 7, 17%) for dinner, or breakfast the day of testing, or 24 h prior. A food recall was obtained in 10 studies (24%). A 3-d food recall was most commonly implemented (4 studies) (39–42), while 4-d (43), 5-d (44), and 7-d (45) food recall were also used.

### Menstrual cycle considerations and oral contraception use

Out of the 16 studies that included female participants, seven studies (44%) did not record any details regarding the menstrual cycle or contraception use. Four studies (25%) reported that the menstrual cycle was not controlled for, but participants were eumenorrheic, while five studies (31%) reported that participants completed the study during the follicular phase or the luteal phase. Five studies (31%) provided details about oral contraception usage. Females in four studies (80%) were not using oral contraception, and one study (20%) examined the differences between females prescribed oral contraception and others who had not used any for at least 12 month (46).

### Protocol characteristics

Across the included studies, there were 21 different metabolic carts used to measure pulmonary gas exchange (see Table 1), while one study used Douglas bags with the Servomex 1400 (Sussex, United Kingdom). The most commonly used metabolic cart (*n* = 9, 21%) was the Oxycon Pro (Jaeger, Würzburg, Germany). There were two types of ergometers used: a cycle ergometer (*n* = 30, 71%) and a treadmill (*n* = 12, 29%). All included studies (with the exception of two (26,44)) were maximal graded exercise tests. The majority of studies (*n* = 26, 62%) performed a single graded exercise test to exhaustion to estimate both outcomes, including LT/GET and Fat_max_. Out of these studies, most performed the graded exercise test to exhaustion (*n* = 10, 38%) with stage intervals of 3 min. Other intervals used included 2-, 4-, 5-, 6-, and 10-min stages. Nine studies (30%) performed 2- 3-, or 5-min stages until the respiratory exchange ratio (RER) >1.0, then all studies increased intensity each minute until exhaustion.

**TABLE 1.** Metabolic carts used during exercise testing across the included studies.

Metabolic Cart	% of Included Studies	Studies
Oxycon Pro (Jaeger, Würzburg, Germany)	21.4	(13,26,27,40,43,47–50)
Oxycon Pro-Delta (Jaeger, Würzburg, Germany)	2.4	(51)
Oxycon Alpha (Jaeger, Würzburg, Germany)	2.4	(15)
Oxycon CPX (Jaeger, Würzburg, Germany)	2.4	(50)
Quark PFT (Cosmed, Rome, Italy)	9.5	(16,52–54)
Quark PFT2 (Cosmed, Rome, Italy)	2.4	(55)
Quark CPET (Cosmed, Rome, Italy)	7.1	(41,50,56)
Quark B2 (Cosmed, Rome, Italy)	2.4	(57)
K5 Portable System (Cosmed, Rome, Italy)	2.4	(24)
Metalyzer (Cortex, Leipzig, Germany)	2.4	(58)
Metalyzer II (Cortex, Leipzig, Germany)	2.4	(25)
Metalyzer 3B (Cortex, Leipzig, Germany)	7.1	(39,42,59)
Metamax 3B (Cortex, Leipzig, Germany)	4.8	(60,61)
Metamax II (Cortex, Leipzig, Germany)	2.4	(62)
Vmax 229 (SensorMedics, CA)	2.4	(14)
2900 Metabolic Cart (SensorMedics, CA)	4.8	(23,63)
CPX Ultima CardiO2 (Medical Graphics Corp, MN)	2.4	(64)
TrueOne 2400 (ParvoMedics, Sandy, UT)	2.4	(65)
Ergocard (Medisoft, Dinant, Belgium)	2.4	(66)
Vyntus CPX (Jaeger, Würzburg, Germany)	2.4	(67)
AE-310S (Minato Medical Science, Osaka, Japan)	2.4	(44)
Douglas bags and Servomex 1400 (Taylor Instrument Analytics, Sussex, United Kingdom)	2.4	(68)
Not recorded	2.4	(69)

Sixteen studies (38%) performed two separate exercise tests, one to identify the $\dot{V}O_{2max}$ of participants followed by a submaximal exercise test to estimate substrate oxidation. Out of these studies, the majority (*n* = 8, 50%) used 1-min stages in the $\dot{V}O_{2max}$ test. Other intervals included 2- or 3-min stages, and two studies performed a ramp protocol. For the additional test to estimate substrate oxidation (i.e., Fat_max_ test), the most commonly used stage length was 6 min (*n* = 5, 31%). Other intervals performed were 2, 3, 5, 6, 9, 10, 15, and 20 min.

### Methods of estimating fat oxidation

There were eight different stoichiometric equations utilized to estimate the carbohydrate and fat oxidation (see Table 2). The equations proposed by Frayn (70) were most commonly used across 20 studies (48%). The methods used to estimate MFO and Fat_max_ varied across the studies (see Table 3). Nine different methods were identified, while two studies did not report how data analysis was performed. The majority of studies (*n* = 24, 57%) used the maximum observed values obtained during the graded exercise tests.

**TABLE 2.** Stoichiometric equations used to estimate fat and carbohydrate oxidation across the included studies.

Stoichiometric Equations	% of Included Studies	Studies
Frayn, 1983 (70) FATox = 1.67 × $\dot{V}O_{2}$ − 1.67 × $\dot{V}CO_{2}$ CHOox = 4.55 × $\dot{V}CO_{2}$ − 3.21 × $\dot{V}O_{2}$	50.0	(13–16,23,25–27,40,43,48,49,52,54,55,57,64,65,67–69,71,72)^*a*^
Jeukendrup and Wallis, 2005 (73) FATox = 1.695 × $\dot{V}O_{2}$ − 1.701 × $\dot{V}CO_{2}$ − 1.77 CHOox: 0%–40% = 4.585 × $\dot{V}CO_{2}$ − 3.226 × $\dot{V}O_{2}$ 40%–50% = 4.344 × $\dot{V}CO_{2}$ − 3.061 × $\dot{V}O_{2}$ 50%–75% = 4.210 × $\dot{V}CO_{2}$ − 2.962 × $\dot{V}O_{2}$	23.8	(24,39,41,44,45,50,56,59,62,74)
Peronnet and Massicotte, 1991 (75) FATox = 1.695 × $\dot{V}O_{2}$ − 1.701 × $\dot{V}CO_{2}$ CHOox = 4.585 × $\dot{V}CO_{2}$ − 3.226 × $\dot{V}O_{2}$	4.8	(46,66)
Elia and Livesey, 1992 (76) FAT% = 1.0 − CHO% CHO% = (5.045 × RQ − 3.582)/(0.36 × RQ + 1.103)	2.4	(53)
Brouwer, 1957 (77) FATox = 1.718 × $\dot{V}O_{2}$ − 1.718 × $\dot{V}O_{2}$ − 0.315 × p (g·min^−1^) Ferrannini, 1988 (78) CHOox: Glucose = 4.55 × $\dot{V}OC_{2}$ − 3.21 × $\dot{V}O_{2}$ (low-medium) Glycogen = 4.09 × $\dot{V}CO_{2}$ − 2.88 × $\dot{V}O_{2}$ (medium-high)	2.4	(47)
Mader’s approach (see Mader (79,80))	2.4	(61)
Calories, CHOox, and FATox were calculated from the steady-state $\dot{V}O_{2}$ adjusted for substrate metabolism using the RER value.	2.4	(63)
No details	11.9	(13,42,58,60,69)

^*a*^References (13,69) cited Consolazio, 1963 (81) and MacRae et al., 1995 (82); however, no equations were provided and it seems as though the equations are identical to Frayn, 1983 (70), but this remains unclear.

CHOox, carbohydrate oxidation; FATox, fat oxidation; p, protein; RQ, respiratory quotient; $\dot{V}CO_{2}$, volume of CO_2_ expired; $\dot{V}O_{2}$, oxygen consumption.

**TABLE 3.** Methods used to estimate MFO and Fat_max_ across the included studies.

Method of Estimation for MFO and Fat_max_	% of Included Studies	Studies
Measured values	57.1	(14,15,23,24,26,27,39,41,43–45,48,52–54,56,57,59,62,63,65,67,71,72)
Third-degree polynomial regression	11.9	(49,50,55,64,74)
Second-degree polynomial regression	7.1	(16,47,68)
Smoothing of the PO vs fat oxidation rate curve	4.8	(46,66)
Sine model (83)	4.8	(13,40)
Two different linear relationships: 1 − RQ and $\dot{V}O_{2}$ vs PO were derived to find the point where the value of the derived equation equals zero	2.4	(69)
Mader’s approach (see Mader (79,80))	2.4	(61)
Highest point of the binomial parabola formed by the fat expenditure-velocity data	2.4	(60)
Visual inspection by two investigators	2.4	(25)
No details	4.8	(42,58)

RQ, respiratory quotient; $\dot{V}O_{2}$, oxygen consumption.

### Methods of estimating the LTs and GETs

Twenty studies used LT as the marker of LT/GET and the methods for determining LT are summarized in Table 4. Twelve different methods were used for estimating LT across these 20 studies. The first increase in lactate concentration above baseline (as described by Coyle et al. (84) and Hagbehg and Coyle (85)) was the most common method of estimation used by six studies (14%). The 13 different referenced methods used to estimate the GET across the 26 studies are summarized in Table 5. Four studies measured both LT and GET.

**TABLE 4.** Definitions and methods used to estimate LT_1_ across the included studies.

LT_1_ Definition and Method of Estimation	% of LT_1_ Studies	Studies
First increase in lactate concentration above baseline (84,85).	14.3	(15,26,27,43,65,66)
Log–log method: The lactate curve was divided into two segments, and the intersection point of the two lines with the lowest residual sum of squares was taken as the LT_1_ (86)	7.1	(67,68,74)
Highest exercise intensity before onset blood lactate concentration (87)	4.8	(16,47)
Calculated using a computer program with the lactate concentration vs workload curve (88)	2.4	(62)
The intersection of blood lactate vs $\dot{V}O_{2}$ (mL·kg^−1^·min^−1^) for both the lower and higher intensities	2.4	(23)
2 mmol·L^−1^ calculated using a polynomial equation (blood lactate concentration vs %$\dot{V}O_{2}$ peak)	2.4	(41)
2 and 4 mmol·L^−1^ calculated using least squares (lactate concentration vs power output)	2.4	(69)
Break point in blood lactate concentration vs power output, representing a departure of blood lactate concentration from a linear baseline, using an R script (Lactate-R) (89)	2.4	(56)
Minimum blood lactate concentration measured during the test + 1.5 mmol·L^−1^ (90)	2.4	(52)
The highest power output and $\dot{V}O_{2}$ obtained just before the curvilinear increase in blood lactate	2.4	(14)
Mader’s approach (see Mader (79,80))	2.4	(61)
Visual inspection	2.4	(50)

LT1, first lactate threshold; $\dot{V}O_{2}$, oxygen consumption.

**TABLE 5.** Definitions and methods used to estimate GET across the included studies.

GET Definition and Method of Estimation	% of GET Studies	Studies
The first maintained rise in VE/$\dot{V}O_{2}$ while VE/$\dot{V}CO_{2}$ remained at a plateau (91)	11.9	(13,24,46,53,60)
Ventilatory equivalent method described by Wasserman et al., 1990 (92)	9.5	(42,58,72,74)
V-slope method (as described by Beaver et al., 1986) (86)	7.1	(40,54,63)
The increase in VE/$\dot{V}O_{2}$ with the absence of changes in the VE/$\dot{V}CO_{2}$ (93)	4.8	(59,64)
Manually identified as the point at which the plot $\dot{V}O_{2}$ vs VE deviated from linearity (94,95)	4.8	(48,49)
The increase in VE/$\dot{V}O_{2}$ and PET_O2_ with the absence of changes in the VE/$\dot{V}CO_{2}$	4.8	(57,66)
The power output where a systematic increase in VE/$\dot{V}O_{2}$ appeared	2.4	(50)
A significant nonlinear increase of VE and VE/$\dot{V}O_{2}$ determined by two independent and experienced investigators (17)	2.4	(63)
Disproportionate increase in expired CO_2_ relative to $\dot{V}O_{2}$ and the VE/$\dot{V}O_{2}$ and VE/$\dot{V}CO_{2}$ (96)	2.4	(25)
VE/$\dot{V}O_{2}$ vs $\dot{V}O_{2}$ was plotted to identify the point during exercise where this curve has a minimum value (97)	2.4	(45)
When the RER rapidly exceeded more than 1.00 and was detectable by breaking the linearity of $\dot{V}CO_{2}$ vs $\dot{V}O_{2}$ curve by the V-slope method (98)	2.4	(44)
Plotted VE and FeO_2_ vs relative $\dot{V}O_{2}$ to identify VT_1_, by identifying the work rate or $\dot{V}O_{2}$ just before the appearance of a nonlinear increase in “IC” in combination with a rapid increase in FeO_2_ and RER. Visual inspection by two investigators	2.4	(23)
The individual anaerobic threshold was determined using a modified V-slope method	2.4	(39)
No definition or method of estimation provided	2.4	(55)

FeO_2_, fraction of expired oxygen; $\dot{V}CO_{2}$, volume of CO2 expired; VE, minute ventilation; VE/$\dot{V}CO_{2}$, ventilatory equivalent for carbon dioxide; VE/$\dot{V}O_{2}$, ventilatory equivalent for oxygen; $\dot{V}O_{2}$, oxygen consumption.

### Risk of bias assessment

**FIGURE 2:**
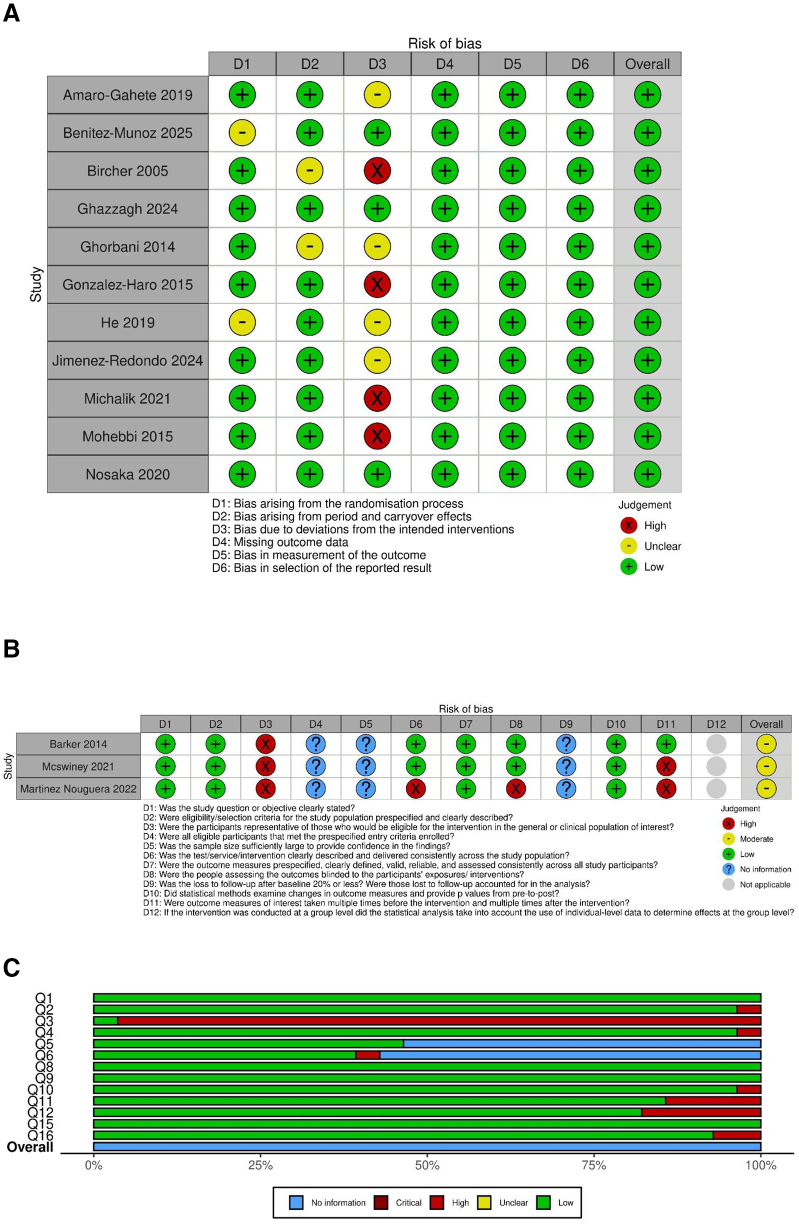
Risk of bias assessments of included studies. A, Cochrane RoB2 for crossover trials. B, National Institute of Health—pre–post studies with no control group. C, Appraisal tool for cross-sectional studies (AXIS).

Based on rating of 766 items across identified studies, interrater reliability for the two reviewers attained a high agreement level (*k* = 0.90, *P* < 0.0001). Risk of bias for all included studies is depicted in Figure 2. All 11 crossover randomized controlled trials had a low risk of bias. While the three pre–post studies had a moderate risk of bias (see Fig. 2C). The cross-sectional tool did not require an overall judgement; however, all 28 studies clearly stated the study aims/objectives. In contrast, sample size was justified in only one study (40). Approximately 65% of the cross-sectional studies did not clearly specify whether the sample was representative of the target population under investigation.

### Fat_max_ versus LTs and GETs

To evaluate the appropriateness of a three-level meta-analysis model compared with a traditional two-level model, an analysis of variance was conducted. The results indicated that the three-level model had lower Akaike Information Criterion and Bayesian Information Criterion values compared with a two-level model (i.e., AIC = 425 vs 513, BIC = 434 vs 519, respectively). Thus, a three-level meta-analysis was the preferred (superior) model. Furthermore, a significant likelihood ratio test (*χ*^2^ = 89.65, *P* < 0.0001) showed the three-level model provided a significant improvement in model fit over the two-level model.

**FIGURE 3:**
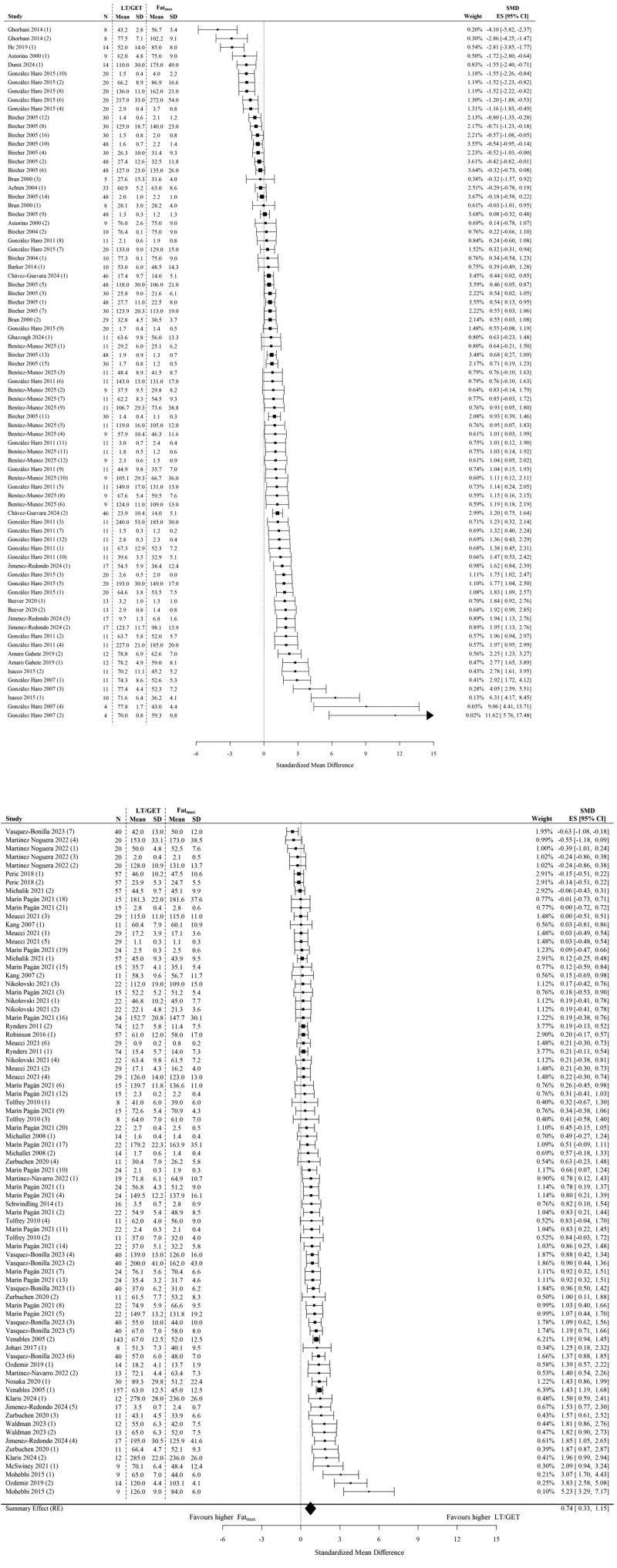
Forest plot for the multilevel meta-analysis random-effects model from included studies that determined AT and Fat_max_ during exercise.

There was a substantially unequal variance across the levels, with 12.3% attributed to level 2 (within studies) and the largest variance of 82.7% attributed to level 3 (between studies). Total heterogeneity was considered high with *I*^2^ = 95.0%. Figure 3 shows the forest plot displaying the multilevel meta-analysis for LT/GET versus Fat_max_. There was a significant difference between the exercise intensities corresponding to the LT/GET and Fat_max_. A large positive ES (ES = 0.74, 95% CI = 0.32–1.16, *P* = 0.0010) was seen, indicating that Fat_max_ typically occurred at a lower exercise intensity than the LT/GET.

Funnel plots are summarized in Supplementary Material 6, Supplemental Digital Content 2, https://links.lww.com/MSS/D420. The Egger’s regression test was nonsignificant (intercept = 1.77, *t* = 1.14, *P* = 0.13), which indicated no potential publication bias. To confirm there was no publication bias, the trim and fill procedure showed no missing studies, indicating the funnel plot was symmetrical, and there were no changes to the overall effect. Sensitivity analyses indicated that the overall ES was robust, with the pooled SMD remaining consistent (0.651–0.807) and all 95% CIs overlapping the main ES, suggesting that no single study disproportionately influenced the meta-analytic results.

### Moderator analysis

A summary of the 23 moderators included in the analysis is provided in Table 6. Among participant characteristics, the ES between Fat_max_ and LT/GET significantly differed from zero in both males (*P* < 0.001) and females (*P* < 0.001), participants of age ≥20 yr (*P* < 0.01), and those with an active to elite fitness level (*P* < 0.05). However, no significant difference was found when sex was unclear, participants were <20 yr, or participants were classified as sedentary. In females, when it was unclear if menstrual cycle or oral contraception was controlled for, Fat_max_ occurred at a lower exercise intensity relative to the LT/GET (*P* < 0.01).

**TABLE 6.** A summary of the protocol characteristic moderator analysis for the multilevel meta-analysis of the markers of LT/GET and Fat_max_.

Moderators	Effects (*k*)	#ES	Overall ES	95% CI	σ^2^ Level 2	σ^2^ Level 3	*F*(df_1_, df_2_)	*P*
Participant characteristics								
Sex					1.552	0.246	*F*(3, 37) = 5.786	0.002**
	Male	103	0.855	0.413 to 1.297*				
	Female	32	0.979	0.493 to 1.464*				
	Not recorded	20	−0.612	−2.171 to 0.947				
	Combined	3	—	—				
Age					1.600	0.242	*F*(3, 39) = 5.508	0.003**
	<20 yr	36	0.206	−0.372 to 0.7833				
	20–29 yr	72	0.943	0.289 to 1.597**				
	≥30 yr	50	0.663	0.296 to 1.030*				
Fitness					1.663	0.245	*F*(4, 38) = 4.480	0.005**
	Elite	85	0.720	0.299 to 1.141**				
	Trained	43	0.600	0.069 to 1.131***				
	Active	17	0.835	0.258 to 1.412**				
	Sedentary	13	0.886	−0.357 to 2.129				
Menstrual cycle					0.922	0.235	*F*(3, 13) = 5.657	0.011***
	Controlled	11	1.370	−0.003 to 2.743				
	Uncontrolled	13	0.326	−0.771 to 1.423				
	Unclear	11	1.099	0.411 to 1.787**				
	N/A	123	—	—				
Oral contraception					0.559	0.220	*F*(2, 14) = 7.979	0.005**
	Excluded	12	0.944	−0.189 to 2.078				
	Unclear	22	0.815	0.326 to 1.305**				
	Included	1	—	—				
	N/A	123	—	—				
Participant acute nutritional status								
Fasting period					1.631	0.239	*F*(3, 39) = 5.503	0.003**
	<6 h	52	0.374	−0.273 to 1.021				
	7–12 h	63	1.069	0.496 to 1.641*				
	Unclear	43	0.538	−0.601 to 1.676				
Regular diet					1.472	0.240	*F*(3, 39) = 8.141	0.0002*
	No	68	1.083	0.376 to 1.791**				
	Yes	38	0.214	−0.476 to 0.904				
	Unclear	52	1.219	0.570 to 1.868*				
Carbohydrate rich					1.663	0.240	*F*(3, 39) = 4.480	0.009**
	No	16	0.348	−0.259 to 0.954				
	Yes	71	0.765	−0.216 to 1.746				
	Unclear	71	0.913	0.317 to 1.509**				
Standardized meals					1.666	0.240	*F*(2, 40) = 6.209	0.005**
	No	131	0.741	0.286 to 1.196**				
	Yes	27	0.745	−0.445 to 1.935				
Food recall					1.663	0.240	*F*(2, 40) = 6.619	0.003**
	No	127	0.763	0.312 to 1.214**				
	Yes	31	0.670	−0.412 to 1.752				
Study design								
Measure					1.676	0.235	*F*(2, 40) = 6.455	0.004**
	GET/VT	80	0.905	0.384 to 1.427*				
	LT	78	0.524	0.020 to 1.029***				
Units					1.631	0.184	*F*(3, 39) = 4.112	0.013***
	$\dot{V}O_{2}$	82	0.747	0.309 to 1.186**				
	PO	37	0.750	0.257 to 1.243**				
	HR	29	0.769	0.280 to 1.258**				
	mmol·L^−1^	8	—	—				
	km·h^−1^	1	—	—				
	SmO_2_%	1	—	—				
Protocol characteristics and equipment								
Mode					1.666	0.240	*F*(2, 40) = 6.128	0.005**
	Cycle ergometer	135	0.748	0.238 to 1.258**				
	Treadmill	23	0.724	−0.061 to 1.509				
Fat_max_ test					1.666	0.240	*F*(2, 40) = 6.177	0.005**
	Combined	123	0.683	0.175 to 1.190**				
	Additional	35	0.841	0.078 to 1.604***				
Constant increments					1.468	0.255	*F*(2, 24) = 4.602	0.020***
	Yes	85	0.693	0.183 to 1.203**				
	No	38	0.654	−0.514 to 1.822				
	N/A	35	—	—				
$\dot{V}O_{2max}$ test protocol					1.994	0.078	*F*(2, 40) = 11.134	0.0001*
	Short	126	0.869	0.404 to 1.333*				
	Long	32	−0.543	−1.468 to 0.383				
Fat_max_ test protocol					2.203	0.077	*F*(2, 40) = 11.314	0.0001*
	Short	103	1.241	0.690 to 1.792*				
	Long	55	−0.108	−0.824 to 0.609				
Indirect calorimetry					1.543	0.242	*F*(5, 36) = 4.903	0.002**
	Breath by breath	90	0.843	0.283 to 1.403**				
	Portable	30	−0.468	−1.720 to 0.784				
	Spirometry	21	1.694	0.354 to 3.034***				
	Mixing chamber	8	0.348	−0.628 to 1.323				
	Multiple	6	1.149	0.299 to 1.999**				
	Unclear	3	—	—				
Analysis methods								
Stoichiometric equations					1.554	0.239	*F*(4, 38) = 5.398	0.002**
	Frayn, 1983	84	0.945	0.484 to 1.407*				
	Jeukendrup and Wallis, 2005	26	0.998	−0.100 to 2.096				
	Alternative equations	19	0.403	−0.892 to 1.697				
	Unclear	29	−0.483	−1.728 to 0.763				
Fat_max_ method					1.786	0.239	*F*(5, 36) = 5.422	0.0008*
	Measured values	80	0.824	0.207 to 1.440***				
	Polynomial	35	1.109	0.555 to 1.664*				
	Sine model	8	0.708	−0.093 to 1.510				
	Other mathematical method	9	0.072	−1.985 to 2.128				
	Unclear	25	0.103	−0.580 to 0.786				
	Visual	1	—	—				
LT_1_ method					0.778	0.401	*F*(4, 13) = 11.565	0.0003*
	First increase above baseline	25	0.624	−0.136 to 1.383				
	Maximal intensity before rise	24	0.461	−0.377 to 1.299				
	Logarithmic/curve fitting	18	0.720	0.471 to 0.970*				
	Fixed lactate concentrations	8	2.001	−0.557 to 4.559				
	Intersection	2	—	—				
	Visual	1	—	—				
	N/A	80	—	—				
GET method					1.946	0.073	*F*(2, 23) = 8.255	0.002**
	Ventilatory equivalents	63	0.439	−0.366 to 1.244				
	V-slope	16	1.442	0.678 to 2.206*				
	No method provided	1	—	—				
	N/A	78	—	—				

* *P* < 0. 001; **P < 0.01, ***P < 0.05.

#ES, number of effect sizes; σ^2^ Level 2, variance between effect sizes; σ^2^ Level 3, variance between studies; df, degrees of freedom; N/A, not applicable; SmO2 %, muscle oxygen saturation percentage; $\dot{V}O_{2}$, oxygen consumption.

Moderator analysis revealed that the participant's acute nutritional status influenced the effect between Fat_max_ and the LT/GET. In studies where participants maintained a regular diet (*n* = 38, ES = 0.214, 95% CI = −0.476 to 0.904, *P* = 0.53), consumed standardized meals (*n* = 27, ES = 0.745, 95% CI = −0.445 to 1.935, *P* = 0.21), completed a food recall (*n* = 31, ES = 0.670, 95% CI = −0.412 to 1.752, *P* = 0.22), or where dietary carbohydrate intake was clearly reported (*n* = 87, ES = 0.348–0.765, 95% CI = −0.259 to 1.746, *P* > 0.05), no significant difference was observed between the exercise intensities at which Fat_max_ and LT/GET occurred. However, when the macronutrient breakdown was unclear (*n* = 71, ES = 0.913, 95% CI = 0.317–1.509, *P* = 0.22), or a fasting period of 7–12 h was implemented (*n* = 63, ES = 1.069, 95% CI = 0.496–1.641, *P* = 0.0005), Fat_max_ occurred at a lower exercise intensity relative to the LT/GET (*P* < 0.01).

When considering study design, Fat_max_ occurred at a lower exercise intensity compared with the LT/GET, regardless of how LT/GET was estimated (GET; *n* = 80, ES = 0.905, *P* < 0.001 or LT; *n* = 78, ES = 0.524, *P* < 0.05), or when the exercise intensities corresponding to Fat_max_ and LT/GET were reported in units of $\dot{V}O_{2}$, power output (PO) or HR (*P* < 0.01).

Various protocol characteristics influenced the ES between Fat_max_ and LT/GET. Fat_max_ occurred at a significantly lower exercise intensity than LT/GET when a cycle ergometer (*n* = 84, ES = 0.945, 95% CI = 0.484–1.407, *P* = 0.0002), a single exercise test (*n* = 123, ES = 0.683, *P* = 0.01), or two separate exercise tests (i.e., a $\dot{V}O_{2max}$ and Fat_max_ test) were used (*n* = 35, ES = 0.841, *P* = 0.03), as well as when protocols included constant workload increments (*P* < 0.01) or short-stage durations (i.e., ≤3 min; *P* < 0.001). This difference was also observed in studies using breath-by-breath indirect calorimetry (*P* < 0.01), spirometry (*P* < 0.05), or multiple calorimeters within a study (*P* < 0.01). In contrast, no significant difference between Fat_max_ and LT/GET was found when treadmill-based protocols were implemented, constant workload increments were not used (e.g., 3-min increments until RER < 1.0, then 1 min until exhaustion), or when long stage durations were performed. Similarly, no significant differences between Fat_max_ and LT/GET were seen when portable indirect calorimeters or mixing chambers were used.

When substrate oxidation was estimated using the stoichiometric equations by Frayn, 1983 (70), Fat_max_ occurred at a significantly lower exercise intensity than LT/GET (*n* = 84, ES = 0.945, 95% CI = 0.484–1.407, *P* = 0.0002). This difference was also evident when MFO and Fat_max_ were derived using measured values or a polynomial regression (*n* = 35, ES = 1.109, 95% CI = 0.555–1.664, *P* = 0.0003), as well as when LT_1_ and GET were determined using logarithmic/curve fitting (*n* = 18, ES = 0.720, 95% CI = 0.471–0.970, *P* < 0.0001) or the V-slope method (*n* = 16, ES = 1.442, 95% CI = 0.678–2.206, *P* = 0.0007), respectively. All other stoichiometric equations, methods to determine Fat_max_, LT, and GET found no significant difference between Fat_max_ and LT/GET (refer to Table 6 for specific analysis methods).

## DISCUSSION

### Summary of key findings

The primary aim of this systematic review and multilevel meta-analysis was to comprehensively search the broader literature and identify whether LT/GET occurred at the same relative exercise intensity as Fat_max_. This review included 42 studies and 158 ES, incorporating 30 additional studies not identified in previous reviews (20,21). Across a large sample including 1505 participants, the primary finding of our review was that Fat_max_ and LT/GET do not occur at the same exercise intensity within the apparently healthy population. Our results showed that Fat_max_ occurred at a lower relative exercise intensity than LT/GET by approximately 5.78% $\dot{V}O_{2max}$ (95% CI = 2.50–9.06), 4.75 mL·min^−1^·kg^−1^ $\dot{V}O_{2}$ (95% CI = 2.05–7.45), 22 W (95% CI = 9–34), or 11 bpm (95% CI = 5–18). Our secondary aim was to explore the potential moderating variables related to participant characteristics, study design, and analytical methods. All 23 moderators assessed had at least one subgroup that significantly influenced the ES between Fat_max_ and LT/GET. These findings highlight the considerable variability in how both Fat_max_ and LT/GET are measured across studies, influenced by factors such as study design, participant preparation, and analysis methods. This variability presents challenges when comparing results across studies or even within individuals pre- and posttesting, for example, if acute nutritional conditions differed between tests. Additionally, our findings contrast the recent meta-analysis by Peric et al. (20), which reported a nonsignificant small effect with no difference between Fat_max_ and the LT/GET (ES= −0.23, 95% CI= −0.56 to 0.09, *P* > 0.05) (20).

### Practical implications

Researchers, clinicians, and coaches should carefully consider study design when using LT/GET as a surrogate for Fat_max_. When considering the population that is being tested, results consistently show that Fat_max_ occurs at a lower exercise intensity than LT/GET in both males and females. This suggests the two thresholds are not equivalent and should not be used interchangeably without careful consideration. In females, the menstrual cycle phase does not appear to influence the exercise intensities Fat_max_ and LT/GET occur at. Although the impact of oral contraceptive use remains unclear, our results indicate that in women not using oral contraception, LT/GET may align with Fat_max._ Only one study included oral contraceptive users; therefore, we could not include this subgroup in the moderator analysis, and future research is warranted. Researchers are recommended to record and report usage within their sample.

We found that Fat_max_ and LT/GET occurred at the same exercise intensity when fasting was <6 h; however, with a 7- to 12-h fast, Fat_max_ occurred at significantly lower intensity than LT/GET. The fasting period in this review ranged from 90 min to an overnight 10- to 12-h fast. Previous research indicates that consuming glucose pre-exercise significantly reduces MFO and Fat_max_ (99–101). Astorino et al. (102) and Croci et al. (103) proposed standardizing diet 48 h pretesting, to increase the reliability of estimating MFO and Fat_max_. In this review, when participants consumed habitual diets, standardized meals, or completed food recalls, alignment between the respective exercise intensities of the two thresholds was found. Based on this evidence, we recommended standardizing nutritional consumption 48 h prior and ensuring an overnight fast (10–12 h) for studies using fasting protocols. Similarly, carbohydrate-rich meals did not appear to affect these intensities. This suggests that pretest meals can be based on individual dietary habits as long as they reflect typical intake. While long-term dietary habits were not assessed in this review, they remain an important topic for future research.

Regardless of whether GET or LT, both differ significantly from Fat_max_. However, the ES was larger when using GET (*P* < 0.001), compared with a moderate ES observed with LT (*P* < 0.05). This suggests that the discrepancy may be more pronounced depending on the LT/GET marker chosen. Protocols using separate tests for $\dot{V}O_{2max}$ and Fat_max_ with stage durations of 4 min or longer tended to produce exercise intensities for LT/GET and Fat_max_ that were comparable. However, limitations in the current literature should be acknowledged. None of the included studies accounted for the mean response time of gas-exchange kinetics, despite using ramp or short-stage protocols. This omission likely contributed to greater divergence between Fat_max_ and LT/GET in studies using short-stage intervals to estimate GET and longer stage intervals to determine Fat_max_. While the choice of an indirect calorimeter often depends on institutional availability, using a portable system or a mixing chamber resulted in no significant differences between the two thresholds. In contrast, breath-by-breath systems—although the most commonly used in the studies reviewed—were associated with Fat_max_ occurring at a significantly lower exercise intensity than LT/GET.

Thirteen different stoichiometric equations were used across studies (Table 2). Prior work has highlighted that such variation limits between study comparisons, with differences of up to 6% for carbohydrate and 3% for fat oxidation rates (26,53,104). Much of this variation reflects whether equations are based on glucose (e.g., Frayn (70))—appropriate at rest—or glycogen, which is predominant during exercise. Consequently, studies using Frayn’s (70) equations showed Fat_max_ occurring at a lower intensity than LT/GET, whereas those using Jeukendrup and Wallis (73) found no difference. Given these systematic differences, and because the Jeukendrup and Wallis (73) equations were developed specifically for exercise and account for substrate shifts across intensities, we recommend their use for exercise-based fat oxidation research.

For estimating MFO and Fat_max,_ a sine model or other mathematical models (see Table 3 for specific methods) reduced the divergence between the thresholds. Although half the studies used measured values, this approach can introduce an error equivalent to the stage increments. Previous research has found the RER method to be less accurate than third-order polynomial or sine curve fitting (83); thus, we recommend the use of either of these methods. The logarithmic or curve fitting method was the only approach for identifying LT that showed a significant difference to Fat_max,_ whereas all other methods showed no significant differences. LT_1_ is often estimated using arbitrary methods, making standard recommendations challenging. These variations should be considered in future research and between study comparisons. Similarly, the V-slope was the only GET method that consistently showed significant differences between Fat_max_ and the LT/GET, and these differences in the GET method need to be carefully considered in future research.

### Physiological Implications

From a physiological perspective, some of the interest in Fat_max_ and LT/GET has been based on the concept that lactate may inhibit fat oxidation. The rapid decrease in fat oxidation at moderate intensities has been linked to the rise in blood lactate concentrations that occurs as a result of an increase in glycolytic flux during moderate exercise (100,105,106). Recently, lactate metabolism has been shown to act as a signaling molecule with hormone-like behaviors and has been referred to as a “lactormone” (107–109). The mechanisms by which lactate inhibits fat oxidation have been investigated in animal models and *in vitro*. Lactate has been shown to limit substrate availability by activating an orphan G-protein coupled receptor (GPR81), which in turn downregulates adipose lipolysis, decreasing circulating free fatty acid concentrations (110,111).

While our findings indicate that Fat_max_ typically occurs below the moderate-to-heavy intensity transition, this temporal sequence does not negate the possibility that lactate contributes to reductions in fat oxidation. Importantly, the moderate-to-heavy intensity transition is defined using blood lactate responses, whereas the proposed inhibitory mechanisms, such as GPR81-mediated suppression of adipose lipolysis, are driven by intramuscular lactate accumulation (110,111). These two signals do not necessarily increase concurrently (107,112). Furthermore, our data cannot determine whether lactate contributes to additional suppression of fat oxidation at intensities above this transition. Therefore, our results suggest that factors other than blood lactate accumulation likely contribute to the initial decline in fat oxidation below the transition, while lactate may still exert additive inhibitory effects at higher intensities.

### Review strengths and limitations

First, this review analyzed a large dataset across 42 studies and 1505 participants. The appropriate use of the multilevel meta-analysis allowed the multiple ES nested within studies to be accounted for, reducing statistical bias and improving estimate precision. Continuous variables such as age were not inputted into the moderator analysis in an attempt to reduce ecological biases. Despite the strengths present within this review, it is not without limitations. One limitation is that the review included only apparently healthy populations, therefore the current findings should not be generalized to populations with clinical conditions, including obesity. The reader is directed to a recent review on optimizing fat oxidation during exercise in obesity (113). The high heterogeneity that remained once looking at subgroups needs to be considered. Due to the inconsistent nature of the approach to measuring Fat_max_ and LT/GET, univariate analysis had to be performed. Covariates could not be inputted together into a single model to provide the effect of all covariates on the ES between Fat_max_ and LT/GET. When considering age, it cannot be confirmed what effect older adults (>55 yr) would have, as there were limited studies that included this age range, so we used the category of >30 yr. Given that our meta-analysis used study-level summary data (means and SD/standard error of the mean), we were unable to assess individual-level agreement between Fat_max_ and LT/GET. Consequently, our results reflect average group-level relationships rather than variability at the individual level.

## CONCLUSIONS

This review found that Fat_max_ occurred at significantly lower exercise intensity relative to the LT/GET. Subsequently, several factors—including participant characteristics, participant preparation, study design, and analytical methods—were shown to influence the magnitude of the difference. Although the overall findings confirm that Fat_max_ occurred at a lower exercise intensity than LT/GET, the review also identified specific methodological conditions under which Fat_max_ may occur at similar exercise intensities as the LT/GET. While our review cannot determine the underlying cause, it is possible that these instances reflect methodological artifacts (e.g., protocol design, stage duration, or threshold determination methods) rather than true physiological alignment. These insights provide important guidance for researchers, clinicians, and coaches aiming to use LT/GET as a surrogate for Fat_max_, particularly in settings where direct measurement of substrate oxidation is not feasible. Standardizing key elements of participant preparation and protocols may help reduce the inconsistency in the current approaches and improve comparability between studies and within participants.

The authors report no conflicts of interests that may have affected the productions and interpretation of this manuscript. The results of the study are presented clearly, honestly, and without fabrication, falsification, or inappropriate data manipulation. The results of the present study do not constitute endorsement by the American College of Sports Medicine.

## Supplementary Material

## References

[1] Achten J Jeukendrup A. Maximal fat oxidation during exercise in trained men. Int J Sports Med. 2003;24(08):603–8. 14598198 10.1055/s-2003-43265

[2] Achten J Gleeson M Jeukendrup AE. Determination of the exercise intensity that elicits maximal fat oxidation. Med Sci Sports Exerc. 2002;34(1):92–7. 11782653 10.1097/00005768-200201000-00015

[3] Romijn J Coyle E Sidossis L. Regulation of endogenous fat and carbohydrate metabolism in relation to exercise intensity and duration. Am J Physiol Endocrinol Metab. 1993;265(3):E380–91. 10.1152/ajpendo.1993.265.3.E380 8214047

[4] Achten J Jeukendrup AE. Optimizing fat oxidation through exercise and diet. Nutrition. 2004;20(7-8):716–27. 15212756 10.1016/j.nut.2004.04.005

[5] Venables MC Jeukendrup AE. Endurance training and obesity: effect on substrate metabolism and insulin sensitivity. Med Sci Sports Exerc. 2008;40(3):495–502. 18379212 10.1249/MSS.0b013e31815f256f

[6] Dumortier M Brandou F Perez-Martin A Fedou C Mercier J Brun J. Low intensity endurance exercise targeted for lipid oxidation improves body composition and insulin sensitivity in patients with the metabolic syndrome. Diabetes Metab. 2003;29(5):509–18. 14631328 10.1016/s1262-3636(07)70065-4

[7] Tan S Wang X Wang J. Effects of supervised exercise training at the intensity of maximal fat oxidation in overweight young women. J Exerc Sci Fit. 2012;10(2):64–9.

[8] Ben Ounis O Elloumi M Makni E. Exercise improves the ApoB/ApoA‐I ratio, a marker of the metabolic syndrome in obese children. Acta Paediatr. 2010;99(11):1679–85. 20594189 10.1111/j.1651-2227.2010.01920.x

[9] Elloumi M Ben Ounis O Makni E Van Praagh E Tabka Z Lac G. Effect of individualized weight‐loss programmes on adiponectin, leptin and resistin levels in obese adolescent boys. Acta Paediatr. 2009;98(9):1487–93. 19489770 10.1111/j.1651-2227.2009.01365.x

[10] Frandsen J Vest SD Larsen S Dela F Helge JW. Maximal fat oxidation is related to performance in an Ironman triathlon. Int J Sports Med. 2017;38(13):975–82. 29050040 10.1055/s-0043-117178

[11] Stisen AB Stougaard O Langfort J Helge JW Sahlin K Madsen K. Maximal fat oxidation rates in endurance trained and untrained women. Eur J Appl Physiol. 2006;98(5):497–506. 17006714 10.1007/s00421-006-0290-x

[12] Chávez-Guevara IA Hernández-Torres RP González-Rodríguez E Ramos-Jiménez A Amaro-Gahete FJ. Biomarkers and genetic polymorphisms associated with maximal fat oxidation during physical exercise: implications for metabolic health and sports performance. Eur J Appl Physiol. 2022;122(8):1773–95. 35362801 10.1007/s00421-022-04936-0

[13] Nikolovski Z Barbaresi S Cable T Peric R. Evaluating the influence of differences in methodological approach on metabolic thresholds and fat oxidation points relationship. Eur J Sport Sci. 2021;21(1):61–8. 31944160 10.1080/17461391.2020.1717640

[14] Rynders CA Angadi SS Weltman NY Gaesser GA Weltman A. Oxygen uptake and ratings of perceived exertion at the lactate threshold and maximal fat oxidation rate in untrained adults. Eur J Appl Physiol. 2011;111(9):2063–8. 21259025 10.1007/s00421-010-1821-z PMC3995406

[15] Achten J Jeukendrup A. Relation between plasma lactate concentration and fat oxidation rates over a wide range of exercise intensities. Int J Sports Med. 2004;25(01):32–7. 14750010 10.1055/s-2003-45231

[16] González-Haro C. Maximal fat oxidation rate and cross-over point with respect to lactate thresholds do not have good agreement. Int J Sports Med. 2011;32(05):379–85. 21380968 10.1055/s-0031-1271763

[17] Wasserman K Whipp BJ Koyl S Beaver WL. Anaerobic threshold and respiratory gas exchange during exercise. J Appl Physiol. 1973;35(2):236–43. 4723033 10.1152/jappl.1973.35.2.236

[18] Robergs RA Ghiasvand F Parker D. Biochemistry of exercise-induced metabolic acidosis. Am J Physiol Regul Integr Comp Physiol. 2004;287(3):R502–16. 15308499 10.1152/ajpregu.00114.2004

[19] Poole DC Rossiter HB Brooks GA Gladden LB. The anaerobic threshold: 50+ years of controversy. J Physiol. 2021;599(3):737–67. 33112439 10.1113/JP279963

[20] Peric R Nikolovski Z Meucci M Tadger P Ferri Marini C Amaro-Gahete FJ. A systematic review and meta-analysis on the association and differences between aerobic threshold and point of optimal fat oxidation. Int J Environ Res Public Health. 2022;19(11):6479. 35682065 10.3390/ijerph19116479 PMC9180269

[21] Ferri Marini C Tadger P Chávez-Guevara IA. Factors determining the agreement between aerobic threshold and point of maximal fat oxidation: follow-up on a systematic review and meta-analysis on association. Int J Environ Res Public Health. 2022;20(1):453. 36612784 10.3390/ijerph20010453 PMC9819531

[22] Jeukendrup A Achten J. Fatmax: a new concept to optimize fat oxidation during exercise? Eur J Sport Sci. 2001;1(5):1–5.

[23] Astorino T. Is the ventilatory threshold coincident with maximal fat oxidation during submaximal exercise in women? J Sports Med Phys Fitness. 2000;40(3):209. 11125763

[24] Meucci M Nandagiri V Kavirayuni VS Whang A Collier SR. Correlation between heart rate at maximal fat oxidation and aerobic threshold in healthy adolescent boys and girls. Pediatr Exerc Sci. 2021;33(3):139–43. 33958504 10.1123/pes.2020-0210

[25] Barker AR Day J Smith A Bond B Williams CA. The influence of 2 weeks of low-volume high-intensity interval training on health outcomes in adolescent boys. J Sports Sci. 2014;32(8):757–65. 24404861 10.1080/02640414.2013.853132

[26] Bircher S Knechtle B Knecht H. Is the intensity of the highest fat oxidation at the lactate concentration of 2 mmol L^−1^? A comparison of two different exercise protocols. Eur J Clin Invest. 2005;35(8):491–8. 16101669 10.1111/j.1365-2362.2005.01538.x

[27] Bircher S Knechtle B. Relationship between fat oxidation and lactate threshold in athletes and obese women and men. J Sports Sci Med. 2004;3(3):174–81. 24482595 PMC3905300

[28] Gmada N Marzouki H Sassi RH. Relative and absolute reliability of the crossover and maximum fat oxidation points and their relationship to ventilatory threshold. Sci Sports. 2013;28(4):e99–e105.

[29] Moher D Shamseer L Clarke M; PRISMA-P Group. Preferred Reporting Items for Systematic Review and Meta-Analysis Protocols (PRISMA-P) 2015 statement. Syst Rev. 2015;4(1):1–9. 25554246 10.1186/2046-4053-4-1 PMC4320440

[30] Heck H Mader A Hess G Mücke S Müller R Hollmann W. Justification of the 4-mmol/l lactate threshold. Int J Sports Med. 1985;6(3):117–30. 4030186 10.1055/s-2008-1025824

[31] Hauser T Adam J Schulz H. Comparison of selected lactate threshold parameters with maximal lactate steady state in cycling. Int J Sports Med. 2014;35(6):517–21. 24227122 10.1055/s-0033-1353176

[32] Veritas Health Innovations. Covidence Systematic Review Software Melbourne, Australia. 2019. Available from: http://www.covidence.org. Accessed July 9, 2022.

[33] Team RC. R: a Language and Environment for Statistical Computing. R Foundation for Statistical Computing; 2023.

[34] Higgins JP Thompson SG. Quantifying heterogeneity in a meta‐analysis. Stat Med. 2002;21(11):1539–58. 12111919 10.1002/sim.1186

[35] Egger M Smith GD Schneider M Minder C. Bias in meta-analysis detected by a simple, graphical test. Br Med J. 1997;315(7109):629–34. 9310563 10.1136/bmj.315.7109.629 PMC2127453

[36] Duval S Tweedie R. Trim and fill: a simple funnel-plot–based method of testing and adjusting for publication bias in meta-analysis. Biometrics. 2000;56(2):455–63. 10877304 10.1111/j.0006-341x.2000.00455.x

[37] Copas J Shi JQ. Meta-analysis, funnel plots and sensitivity analysis. Biostatistics. 2000;1(3):247–62. 12933507 10.1093/biostatistics/1.3.247

[38] Kaminsky LA Arena R Myers J., editors. Updated reference standards for cardiorespiratory fitness measured with cardiopulmonary exercise testing: data from the Fitness Registry and the Importance of Exercise National Database (FRIEND). In: Mayo Clinic Proceedings. Elsevier; 2022. 10.1016/j.mayocp.2021.08.020 34809986

[39] Mohebbi H Nourshahi M Ghasemikaram M Safarimosavi S. Effects of exercise at individual anaerobic threshold and maximal fat oxidation intensities on plasma levels of nesfatin-1 and metabolic health biomarkers. J Physiol Biochem. 2015;71(1):79–88. 25637303 10.1007/s13105-015-0383-2

[40] Zurbuchen A Lanzi S Voirol L. Fat oxidation kinetics is related to muscle deoxygenation kinetics during exercise. Front Physiol. 2020;11:571. 32581846 10.3389/fphys.2020.00571 PMC7289152

[41] McSwiney FT Fusco B McCabe L. Changes in body composition and substrate utilization after a short-term ketogenic diet in endurance-trained males. Biol Sport. 2021;38(1):145–52. 33795923 10.5114/biolsport.2020.98448 PMC7996378

[42] Martínez Noguera FJ Marín-Pagán C Chung LH Alcaraz PE. Biomarker changes in oxygen metabolism, acid-base status, and performance after the off-season in well-trained cyclists. Nutrients. 2022;14(18):3808. 36145185 10.3390/nu14183808 PMC9506402

[43] Robinson S Chambers E Fletcher G Wallis G. Lipolytic markers, insulin and resting fat oxidation are associated with maximal fat oxidation. Int J Sports Med. 2016;37(08):607–13. 27116342 10.1055/s-0042-100291

[44] Nosaka N Tsujino S Honda K Suemitsu H Kato K. Enhancement of fat oxidation during submaximal exercise in sedentary persons: variations by medium‐chain fatty acid composition and age group. Lipids. 2020;55(2):173–83. 32058596 10.1002/lipd.12222

[45] Ghorbani S Mohebbi H Safarimosavi S Ghasemikaram M. The effect of different recovery methods on blood lactate removal in wrestlers. J Sports Med Phys Fitness. 2014;55(4):273–9. 25412604

[46] Isacco L Thivel D Pereira B Duclos M Boisseau N. Maximal fat oxidation, but not aerobic capacity, is affected by oral contraceptive use in young healthy women. Eur J Appl Physiol. 2015;115(5):937–45. 25519952 10.1007/s00421-014-3075-7

[47] González-Haro C. Differences in physiological responses between short- vs. long-graded laboratory tests in road cyclists. J Strength Cond Res. 2015;29(4):1040–8. 25330085 10.1519/JSC.0000000000000741

[48] Venables MC Achten J Jeukendrup AE. Determinants of fat oxidation during exercise in healthy men and women: a cross-sectional study. J Appl Physiol. 2005;98(1):160–7. 15333616 10.1152/japplphysiol.00662.2003

[49] Martinez-Navarro I Montoya-Vieco A Collado E Hernando B Hernando C. Ultra trail performance is differently predicted by endurance variables in men and women. Int J Sports Med. 2020;43(07):600–7. 33017851 10.1055/a-1255-3083

[50] Klaris MB Cubel C Bruun TR. Performance and fatigue patterns in elite cyclists during 6 h of simulated road racing. Scand J Med Sci Sports. 2024;34(7):e14699. 39011951 10.1111/sms.14699

[51] Isacco L Thivel D Pelle AM. Oral contraception and energy intake in women: impact on substrate oxidation during exercise. Appl Physiol Nutr Metab. 2012;37(4):646–56. 22607658 10.1139/h2012-031

[52] González-Haro C Galilea PA Gonzáález-de-Suso JM Drobnic F Escanero JF. Maximal lipidic power in high competitive level triathletes and cyclists. Br J Sports Med. 2007;41(1):23–8. 17062656 10.1136/bjsm.2006.029603 PMC2465139

[53] Peric R Meucci M Bourdon PC Nikolovski Z. Does the aerobic threshold correlate with the maximal fat oxidation rate in short stage treadmill tests? J Sports Med Phys Fitness. 2017;58(10):1412–7. 28745473 10.23736/S0022-4707.17.07555-7

[54] Özdemir C Özgünen K Günaşti O Eryilmaz S Kilci A Kurdak S. Changes in substrate utilization rates during 40 min of walking within the Fat_max_ range. Physiol Int. 2019;106(3):294–304. 31560234 10.1556/2060.106.2019.28

[55] Johari HM Caszo BA Knight VF Lumley SA Hamid AKA Gnanou JV. Maximal oxygen uptake, ventilatory threshold and fat oxidation after an incremental exercise test in triathletes. Gazz Med Ital Arch Sci Med. 2017;176(12):640–6.

[56] Beever AT Tripp TR Zhang J MacInnis MJ. NIRS-derived skeletal muscle oxidative capacity is correlated with aerobic fitness and independent of sex. J Appl Physiol. 2020;129(3):558–68. 32702279 10.1152/japplphysiol.00017.2020 PMC7517427

[57] Michalik K Danek N Zatoń M. Comparison of the RAMP and Step Incremental Exercise Test protocols in assessing the maximal fat oxidation rate in youth cyclists. J Hum Kinet. 2021;80(1):163–72. 34868426 10.2478/hukin-2021-0104 PMC8607772

[58] Marín-Pagán C Dufour S Freitas TT Alcaraz PE. Performance profile among age categories in young cyclists. Biology. 2021;10(11):1196. 34827189 10.3390/biology10111196 PMC8614687

[59] Bonilla AV González-Custodio A Timón R Cardenosa A Camacho-Cardenosa M Olcina G. Training zones through muscle oxygen saturation during a graded exercise test in cyclists and triathletes. Biol Sport. 2022;40(2):439–48. 37077776 10.5114/biolsport.2023.114288 PMC10108753

[60] He Z Tian Y Valenzuela PL. Myokine/adipokine response to “aerobic” exercise: is it just a matter of exercise load? Front Physiol. 2019;10:691. 31191366 10.3389/fphys.2019.00691 PMC6549222

[61] Dunst AK Hesse C Ueberschär O. Understanding optimal cadence dynamics: a systematic analysis of the power-velocity relationship in track cyclists with increasing exercise intensity. Front Physiol. 2024;15:1343601. 38645689 10.3389/fphys.2024.1343601 PMC11027132

[62] Schwindling S Scharhag-Rosenberger F Kindermann W Meyer T. Limited benefit of Fat_max_-test to derive training prescriptions. Int J Sports Med. 2013;35(04):280–5. 24022578 10.1055/s-0033-1349106

[63] Kang J Hoffman JR Ratamess NA Faigenbaum AD Falvo M Wendell M. Effect of exercise intensity on fat utilization in males and females. Res Sports Med. 2007;15(3):175–88. 17987506 10.1080/15438620701525474

[64] Amaro-Gahete FJ Jurado-Fasoli L Triviño AR Sanchez-Delgado G Helge JW Ruiz JR. Diurnal variation of maximal fat-oxidation rate in trained male athletes. Int J Sports Physiol Perform. 2019;14(8):1140–6. 30702364 10.1123/ijspp.2018-0854

[65] Waldman HS Bryant AR Knight SN. Assessment of metabolic flexibility by substrate oxidation responses and blood lactate in women expressing varying levels of aerobic fitness and body fat. J Strength Cond Res. 2023;37(3):581–8. 35836305 10.1519/JSC.0000000000004316

[66] Michallet A-S Tonini J Regnier J. Methodological aspects of crossover and maximum fat-oxidation rate point determination. Diabetes Metab. 2008;34(5):514–23. 18823806 10.1016/j.diabet.2008.04.004

[67] Benítez-Muñoz JA Guisado-Cuadrado I Rojo-Tirado MA. Females have better metabolic flexibility in different metabolically challenging stimuli. Appl Physiol Nutr Metab. 2024;50:1–12. 10.1139/apnm-2024-0217 39437435

[68] Tolfrey K Jeukendrup AE Batterham AM. Group- and individual-level coincidence of the ‘Fat_max_’ and lactate accumulation in adolescents. Eur J Appl Physiol. 2010;109(6):1145–53. 20376480 10.1007/s00421-010-1453-3

[69] Brun J Bouchahda C Chaze D Aïssa Benhaddad A Micallef J Mercier J. The paradox of hematocrit in exercise physiology: which is the “normal” range from an hemorheologist’s viewpoint? Clin Hemorheol Microcirc. 2000;22(4):287–303. 11081466

[70] Frayn K. Calculation of substrate oxidation rates in vivo from gaseous exchange. J Appl Physiol. 1983;55(2):628–34. 6618956 10.1152/jappl.1983.55.2.628

[71] Ghazzagh A Naderi A Agha-Alinejad H. Acute effects of taurine supplementation on maximal fat oxidation and FATmax in recreational endurance runners: a randomised, placebo-controlled, crossover, and triple-blinded study. Int J Sport Nutr Exerc Metab. 2024;35(1):3–11. 39419489 10.1123/ijsnem.2024-0076

[72] Jiménez-Redondo G Castro-Frecha B Martínez-Noguera FJ Alcaraz PE Marín-Pagán C. Physiological responses in trail runners during a maximal test with different weighted-vest loads. Sports (Basel). 2024;12(7):189. 39058080 10.3390/sports12070189 PMC11280601

[73] Jeukendrup A Wallis G. Measurement of substrate oxidation during exercise by means of gas exchange measurements. Int J Sports Med. 2005;26(S 1):S28–37. 15702454 10.1055/s-2004-830512

[74] Chávez-Guevara IA González-Rodríguez E Moreno-Brito V. The polymorphism T1470A of the *SLC16A1* gene is associated with the lactate and ventilatory thresholds but not with fat oxidation capacity in young men. Eur J Appl Physiol. 2024;124(6):1835–43. 38216723 10.1007/s00421-023-05407-w

[75] Peronnet F Massicotte D. Table of nonprotein respiratory quotient: an update. Can J Sport Sci. 1991;16(1):23–9. 1645211

[76] Elia M Livesey G. Energy expenditure and fuel selection in biological systems: the theory and practice of calculations based on indirect calorimetry and tracer methods. World Rev Nutr Diet. 1992;70:68–131. 1292242 10.1159/000421672

[77] Brouwer E. On simple formulae for calculating the heat expenditure and the quantities of carbohydrate and fat oxidized in metabolism of men and animals, from gaseous exchange (oxygen intake and carbonic acid output) and urine-N. Acta Physiol Pharmacol Neerl. 1957;6:795–802. 13487422

[78] Ferrannini E. The theoretical bases of indirect calorimetry: a review. Metabolism. 1988;37(3):287–301. 3278194 10.1016/0026-0495(88)90110-2

[79] Mader A. Eine theorie zur berechnung der dynamik und des steady state von phosphorylierungszustand und stoffwechselaktivität der muskelzelle als folge des energiebedarfs. na; 1984.

[80] Mader A. Glycolysis and oxidative phosphorylation as a function of cytosolic phosphorylation state and power output of the muscle cell. Eur J Appl Physiol. 2003;88(4-5):317–38. 12527960 10.1007/s00421-002-0676-3

[81] Consolazio CF. Physiological measurements of metabolic functions in man. In: The Computation of Metabolic Balances. 1963. p. 313–317.

[82] MacRae HS-H Noakes TD Dennis SC. Role of decreased carbohydrate oxidation on slower rises in ventilation with increasing exercise intensity after training. Eur J Appl Physiol Occup Physiol. 1995;71(6):523–9. 8983920 10.1007/BF00238555

[83] Cheneviere X Malatesta D Peters EM Borrani F. A mathematical model to describe fat oxidation kinetics during graded exercise. Med Sci Sports Exerc. 2009;41(8):1615–25. 19568198 10.1249/MSS.0b013e31819e2f91

[84] Coyle E Martin W Ehsani A. Blood lactate threshold in some well-trained ischemic heart disease patients. J Appl Physiol. 1983;54(1):18–23. 6826403 10.1152/jappl.1983.54.1.18

[85] Hagbehg JM Coyle EF. Physiological determinants of endurance performance as studied in colnpetilive racewalkers. Med Sci Sports Exerc. 1983;15(4):287–9. 6621317 10.1249/00005768-198315040-00006

[86] Beaver WL Wasserman K Whipp BJ. A new method for detecting anaerobic threshold by gas exchange. J Appl Physiol. 1986;60(6):2020–7. 3087938 10.1152/jappl.1986.60.6.2020

[87] Davis JA Vodak P Wilmore JH Vodak J Kurtz P. Anaerobic threshold and maximal aerobic power for three modes of exercise. J Appl Physiol. 1976;41(4):544–50. 985399 10.1152/jappl.1976.41.4.544

[88] Stegmann H Kindermann W Schnabel A. Lactate kinetics and individual anaerobic threshold. Int J Sports Med. 1981;2(3):160–5. 7333753 10.1055/s-2008-1034604

[89] Newell J Higgins D Madden N. Software for calculating blood lactate endurance markers. J Sports Sci. 2007;25(12):1403–9. 17786693 10.1080/02640410601128922

[90] Dickhuth H-H Huonker M Münzel T Drexler H Berg A Keul J. Individual anaerobic threshold for evaluation of competitive athletes and patients with left ventricular dysfunction. In: Advances in Ergometry. Springer; 1991. p. 173–179.

[91] Meyer T Lucia A Earnest CP Kindermann W. A conceptual framework for performance diagnosis and training prescription from submaximal gas exchange parameters-theory and application. Int J Sports Med. 2005;26(Suppl 1):S38–48. 15702455 10.1055/s-2004-830514

[92] Wasserman K Beaver WL Whipp BJ. Gas exchange theory and the lactic acidosis (anaerobic) threshold. Circulation. 1990;81(1 Suppl):II14–30. 2403868

[93] Lucía A Hoyos J Chicharro JL. The slow component of VO2 in professional cyclists. Br J Sports Med. 2000;34(5):367–74. 11049147 10.1136/bjsm.34.5.367 PMC1756236

[94] McLellan T Skinner J. Blood lactate removal during active recovery related to the aerobic threshold. Int J Sports Med. 1982;03(04):224–9.

[95] Skinner JS McLellan TH. The transition from aerobic to anaerobic metabolism. Res Q Exerc Sport. 1980;51(1):234–48. 7394286 10.1080/02701367.1980.10609285

[96] Wasserman K Hansen J Sue D Stringer W Whipp BJ. Principles of Exercise Testing and Interpretation: Including Pathophysiology and Clinical Applications. 4th ed. Lippincott Williams & Wilkins; 2005.

[97] Hollmann W. 42 years ago—development of the concepts of ventilatory and lactate threshold. Sports Med. 2001;31(5):315–20. 11347682 10.2165/00007256-200131050-00002

[98] Schneider DA Phillips SE Stoffolano S. The simplified V-slope method of detecting the gas exchange threshold. Med Sci Sports Exerc. 1993;25(10):1180–4. 8231764

[99] Achten J Jeukendrup AE. The effect of pre-exercise carbohydrate feedings on the intensity that elicits maximal fat oxidation. J Sports Sci. 2003;21(12):1017–24. 14748459 10.1080/02640410310001641403

[100] Coyle EF Jeukendrup AE Wagenmakers A Saris W. Fatty acid oxidation is directly regulated by carbohydrate metabolism during exercise. Am J Physiol Endocrinol Metab. 1997;273(2):E268–75. 10.1152/ajpendo.1997.273.2.E268 9277379

[101] Horowitz JF Mora-Rodriguez R Byerley LO Coyle EF. Lipolytic suppression following carbohydrate ingestion limits fat oxidation during exercise. Am J Physiol. 1997;273(4):E768–75. 9357807 10.1152/ajpendo.1997.273.4.E768

[102] Astorino TA Edmunds RM Clark A. Change in maximal fat oxidation in response to different regimes of periodized high-intensity interval training (HIIT). Eur J Appl Physiol. 2017;117(4):745–55. 28251399 10.1007/s00421-017-3535-y

[103] Croci I Borrani F Byrne N. Reproducibility of Fatmax and fat oxidation rates during exercise in recreationally trained males. PLoS One. 2014;9(6):e97930. 24886715 10.1371/journal.pone.0097930 PMC4041727

[104] Maunder E Plews DJ Kilding AE. Contextualising maximal fat oxidation during exercise: determinants and normative values. Front Physiol. 2018;9:599. 29875697 10.3389/fphys.2018.00599 PMC5974542

[105] Sidossis LS Gastaldelli A Klein S Wolfe RR. Regulation of plasma fatty acid oxidation during low- and high-intensity exercise. Am J Physiol Endocrinol Metab. 1997;272(6):E1065–70. 10.1152/ajpendo.1997.272.6.E1065 9227453

[106] San-Millán I Brooks GA. Assessment of metabolic flexibility by means of measuring blood lactate, fat, and carbohydrate oxidation responses to exercise in professional endurance athletes and less-fit individuals. Sports Med. 2018;48(2):467–79. 28623613 10.1007/s40279-017-0751-x

[107] Brooks GA. Cell–cell and intracellular lactate shuttles. J Physiol. 2009;587(Pt 23):5591–600. 19805739 10.1113/jphysiol.2009.178350 PMC2805372

[108] Brooks G. Lactate shuttles in nature. Biochem Soc Trans. 2002;30(2):258–64. 12023861 10.1042/

[109] Hashimoto T Hussien R Oommen S Gohil K Brooks GA. Lactate sensitive transcription factor network in L6 cells: activation of MCT1 and mitochondrial biogenesis. FASEB J. 2007;21(10):2602–12. 17395833 10.1096/fj.07-8174com

[110] Liu C Wu J Zhu J. Lactate inhibits lipolysis in fat cells through activation of an orphan G-protein-coupled receptor, GPR81. J Biol Chem. 2009;284(5):2811–22. 19047060 10.1074/jbc.M806409200

[111] Cai T-Q Ren N Jin L. Role of GPR81 in lactate-mediated reduction of adipose lipolysis. Biochem Biophys Res Commun. 2008;377(3):987–91. 18952058 10.1016/j.bbrc.2008.10.088

[112] Jorfeldt L Juhlin-Dannfelt A Karlsson J. Lactate release in relation to tissue lactate in human skeletal muscle during exercise. J Appl Physiol Respir Environ Exerc Physiol. 1978;44(3):350–2. 632175 10.1152/jappl.1978.44.3.350

[113] Chavez-Guevara IA Amaro-Gahete FJ Ramos-Jimenez A Brun JF. Toward exercise guidelines for optimizing fat oxidation during exercise in obesity: a systematic review and meta-regression. Sports Med. 2023;53(12):2399–416. 37584843 10.1007/s40279-023-01897-y

